# When Violence Can Appear With Different Male Partners: Identification of Resilient and Non-resilient Women in the European Union

**DOI:** 10.3389/fpsyg.2018.00877

**Published:** 2018-06-01

**Authors:** Juan Herrero, Pep Vivas, Andrea Torres, Francisco J. Rodríguez

**Affiliations:** ^1^Department of Psychology, University of Oviedo, Oviedo, Spain; ^2^Department of Psychology, Universitat Oberta de Catalunya, Barcelona, Spain

**Keywords:** intimate partner violence, resiliency, European Union, multilevel, child abuse, adult victimization, victimization by multiple male partners

## Abstract

**Introduction:** Little scholarly attention has been paid to the analysis of the history of intimate partner violence (IPV) against women with different male partners and how it could be related to levels of IPV with the current male partner. From this point of view, been a victim of IPV could increase the vulnerability of women and, therefore, exert a negative influence on the selection of partners over time, thus increasing the odds of potentially mating with abusive male partners. Alternatively, for some women victims of IPV in previous relationships, there may be additional resources that reduce their vulnerability to victimization by new partners.

**Methodology:** The present study analyzes levels of IPV in different partners of 2376 heterosexual women from the 28 countries of the European Union living together as a couple who had previously lived with a different male partner.

**Analysis/Discussion:** Multilevel regression results indicated that resilient women were younger, more satisfied with household income, and were involved in shorter relationships. As for their previous levels of victimization, they scored lower on child abuse and non-partner adult victimization. Also, their levels of victimization from previous partners were the same as those of the non-resilient women, with the exception of physical IPV victimization where resilient women scored higher than non-resilient women. Resilient women also informed the interviewer to have ended the abusive relationship because of the violence to a greater extent than non-resilient women and seemed to suffer fewer psychological difficulties due to previous violent relationships. Finally, countries scoring higher on human development index (HDI) showed a larger proportion of resilient women.

**Conclusion:** Resilient women are mostly characterized by fewer psychological difficulties and lower frequency of adverse situations (in childhood or in adulthood) when compared to non-resilient women. Although resilient women reported a higher physical IPV, they nevertheless show fewer psychological sequelae and a greater ability to end abusive relationships. In addition, the human development of the countries in which they live also seems to reinforce their resilience, which suggests combining intervention policies at the individual and contextual levels.

## Introduction

Research on intimate partner violence (IPV) against women has provided abundant empirical evidence on its main risk factors. However, less scholarly attention has been paid to the analysis of the history of IPV with different male partners and how it could be related to levels of IPV with the current male partner. Using data from 2376 women of the European Union that had suffered IPV from previous partners and were currently living with a new partner, we analyzed the characteristics of those women who informed of no IPV with their current partner as compared to those women who reported IPV with both previous and current partners.

Despite the growing interest that the study of IPV has generated in the last decades, a minor research effort has been dedicated to the study of the relationship between current IPV and the previous history of IPV. According to a recent review of studies conducted by Ørke et al. (2018) on risk for victimization of IPV by multiple partners the most striking finding was the scarcity of studies on this topic. Improving our understanding of why some women are victimized by multiple partners while other victims seem to be able to create new intimate relationships free of IPV is a promising field of research.

Although a percentage of women are victimized again by different partners (Bybee and Sullivan, 2002; Cattaneo and Goodman, 2005; Stein et al., 2016), some victims of IPV seem to be resilient to the negative effects of abuse (DuMont et al., 2007). This resiliency might be viewed as the ability to achieve good developmental outcomes while experiencing negative circumstances that pose a risk to normal development (Masten, 1994). Among those women who have suffered IPV with previous partners, those who now maintain relationships free of IPV with their current partners can be considered resilient women to the IPV. This invites to deepen the analysis of the characteristics of resilient women. The literature on this topic is very scarce and the available evidence does not yet provide a clear picture of the risk and protective factors of IPV revictimization by different partners. We review the empirical evidence available on this topic.

One of the aspects in which there seems to be greater agreement among researchers is that adverse experiences during childhood can have long-term effects in adult relationships. There is empirical evidence coming from both retrospective and prospective studies that victims of child abuse are somewhat predisposed to also be victims of IPV in their adulthood (see Herrero et al., 2018 for an analysis). As Ørke et al. (2018) have noted in their review of studies, women with IPV by multiple partners use to be exposed to more types of childhood violence and sexual abuse than women exposed to IPV by one partner. According to this, adverse experiences during childhood not only would predict higher IPV levels in adulthood but also a greater propensity to be victimized by different male partners.

Another set of IPV revictimization risk factors includes the type, frequency, and severity of abuse suffered in previous intimate relationships (Kuijpers et al., 2011). There are studies that have found that victims of more severe IPV tend to be victimized in other intimate relationships (Testa et al., 2003; Cole et al., 2008). Kuijpers et al. (2011) have used Foa et al. (2000) model to explain this association: partner violence causes psychological difficulties that, in turn, put women at greater risk of revictimization by hindering the victim’s ability to curtail future violence. According to this, women who experience more severe IPV, as well as higher levels of psychological difficulties, become more vulnerable, thus increasing the odds of being victimized again by new partners.

Having been victimized in adulthood by people other than the male partner or the ex-partner has also been linked to IPV revictimization of women (Nishith et al., 2000; Stein et al., 2016). There are studies that suggest that some IPV victimization may arise from previous victimization experiences that cause interpersonal difficulties with, in turn, increase the risk of revictimization (Cole et al., 2008). Although most of this research is based on victims of child abuse (see Herrero et al., 2018 for an analysis), it is also possible than non-victims of abuse might also generate this type of social environment (Cole et al., 2008).

Staying in an abusive relationship has been linked to an increase in IPV (Fleury et al., 2000). Ending the relationship with the abuser may be difficult for the victim (DeKeseredy et al., 2017; Edwards et al., 2018) – if, for example, an increase in violence is anticipated as a consequence. As it has been pointed out, however, the end of the relationship may be a protective factor for future episodes of IPV (Grasley et al., 1999) that has been related to both post-traumatic growth and an increase in psychosocial resources (Senter and Caldwell, 2002; Cobb et al., 2006). The literature on victimization by multiple partners has rarely paid attention to this fact and little is known about the potential effect that ending a violent relationship could have on the creation of new relationships free of IPV. Those women who break their violent relationship can be empowered to create other intimate relationships free of violence, thus reducing the likelihood of IPV with new partners. As a result, breaking the violent relationship would be a protective factor while staying in the violent relationship would be a risk factor for victimization by multiple partners. In relation to this, the presence of children who are witnesses of the IPV could be an important factor in explaining the decisions of the victims of IPV on the end of the violent relationship and also on their resilience to the IPV. As Rhodes et al. (2010) have suggested, mothers may wish to protect their children from harmful effects of violence but may want to keep the family together, thus avoiding any instability caused by legal system involvement. There is no empirical evidence that one or the other decision is more related to the resilience of women, despite the fact that negative consequences of the continued exposure of children to episodes of IPV have been recognized in the literature (Bogat et al., 2006).

Another set of risk factors identified by Ørke et al. (2018) in their review of studies is the characteristics of women and/or the relationship. Although the evidence is not conclusive, it seems that the youngest women are more at risk of being victimized by multiple partners (Testa et al., 2003; Alexander, 2009). Other sociodemographic characteristics such as income, education, ethnicity, or unemployment, seem to be unrelated to victimization by multiple partners (see Ørke et al., 2018) although there is empirical evidence of their relationships with IPV (Herrero et al., 2016, 2017b). Length of the relationship with a new partner seems also to be positively related to IPV with a new partner (Cole et al., 2008).

While the aspects related to victims and their relationships, although in a limited way, have received the attention of researchers, the study of structural influences in the IPV revictimization process has traditionally been neglected. There is currently a growing body of empirical evidence that links the existence of IPV with some structural conditions whose influence goes beyond the individual characteristics (Falb et al., 2015; Heise and Kotsadam, 2015). This evidence does not seem to have been investigated in studies on IPV revictimization, which could reflect a potential limitation of research in this area. Structural aspects such as the human development of a country have shown to be predictors of the country’s IPV levels, after controlling for a wide range of individual factors (Herrero et al., 2017b, 2018). It would be necessary to verify if these structural risk factors also play a relevant role in the IPV revictimization of women, especially in multi-country studies.

### The Present Study

The study of victimization by multiple partners has been a relatively neglected topic in IPV research against women. In recent years, however, there has been a growing interest in knowing why some women who have suffered IPV are able to create new intimate relationships free of IPV. The literature has identified some characteristics of these resilient women: they suffered less child abuse, they have been less victimized in their adulthood (by the partner or ex-partner or by other adults), the consequences of IPV were less severe (fewer psychological difficulties), their new relationships are shorter, and tend to be older. The available evidence, however, is not conclusive (Ørke et al., 2018) and, therefore, a greater research effort is needed to clarify both the risk factors and the protective factors. This lack of conclusive empirical evidence is partially explained by the great diversity of samples used in the investigations, sometimes of a small size and limited representativeness, which limits the generalizability of the results. Also, most of the research in this area explores some of the protective and risk factors, but rarely consider multiple factors in a single research design. An additional research effort should be directed toward the evaluation of integrative models and at the same time to favor the use of large samples with increasing degree of representativeness.

Taking into account all the above, the present study aims to analyze the differences between women who suffered IPV with previous partners and also suffer IPV with their current partner (non-resilient women) and women who have suffered IPV with previous partners and do not experience IPV with her current partner (resilient women). The study was carried out in a large sample of women from the 28 member countries of the European Union (*N* = 2376) from a probabilistic sample of 42,000 women.

Based on the theory and empirical evidence available, the study includes the evaluation of various aspects potentially related to IPV victimization by multiple partners. It includes not only a set of victimization variables from partners, ex-partners, and other adults, but also experiences of child abuse and other risk factors such as psychological difficulties, length of relationships, children witnessing partner abuse, or being able to end a previous relationship because of the violence. It also incorporates a measure of the human development of the country in which the victim lives. Sociodemographic variables such as age, education, income, and size of locality were also included in the study since there are previous research linking these sociodemographic characteristics with IPV from both single and multiple partners (Kuijpers et al., 2011; Palmetto et al., 2013; Ørke et al., 2018). Finally, to better control for potentially biased responses, lack of sincerity of the respondent as appraised by the interviewer was also taken into account.

## Materials and Methods

### Participants

Data from the survey on women’s well-being and safety conducted in Europe in 2012 in all the 28 member states of the European Union, were used for this study. The survey was conducted by professional interviewers, trained to guarantee confidentiality. Participation was voluntary and at any time during the interview, the respondent could leave the interview if she did not wish to continue. All the information that allowed to identify the respondents was eliminated from the database. In each Member State, the survey covered all women aged 18–74 years. Certain populations were excluded from the study as people living in institutions and homeless people. Family members who were away from home for a period of 3 months or more were also excluded from the selection. The interviewers presented the survey as a study on women’s welfare and safety. Once the respondent accepted to participate, more details about the content of the survey were provided. This was done to protect the respondent’s safety in case she lived with someone who did not want the respondent to participate in a survey on violence against women, including possible perpetrators of violence. The averaged response rates for all countries was 77% (FRA, European Union Agency for Fundamental Rights, 2014). We used data from 2376 heterosexual women living with a male partner who had also previously lived with a different male partner and suffered IPV with a previous partner.

### Measures

#### Outcome Variable

##### Resiliency

Two groups of women were formed based on their IPV scores with previous and current partners (see below). A first group of women who reported to the interviewer having suffered IPV only with previous partners but not with the current partner (*n* = 1624, 72.7%) (*only previous IPV group*); and a second group of women who reported to the interviewer having suffered IPV with both previous and current partners (*n* = 594, 23.7%) (*previous and current IPV group*). Women in the first group (*only previous IPV group*) were considered to be resilient (resilient = 1) whereas women in the second group (*previous and current IPV group*) were considered to be non-resilient (resilient = 0).

#### Previous Relationships

##### IPV with the previous partner

Respondents informed to the interviewer whether they had experienced psychological, physical, or sexual violence with a previous partner (different from the actual partner).

##### Physical IPV

Respondents were asked how often their previous partner had used physical violence toward them across five items (pushed them, slapped them, thrown hard objects at them, grabbed or pulled their hair, beat them with a fist or a hard object or kicked them). Category responses ranged from 1 (*never*) to 4 (*6 or more times*). Average physical violence from previous partner was *M* = 1.32 (SD = 0.64) (Cronbach’s α = 0.88).

##### Psychological IPV

Respondents were asked how often their previous partner had used psychological violence across four items (belittled or humiliated them in front of other people or in public, scared or intimidated them on purpose, made them watch pornographic material against their wishes, or threatened to hurt them physically). Category responses were coded 1 (*No*) to 2 (*Yes*). Average psychological violence from previous partner was *M* = 1.22 (SD = 0.31) (Cronbach’s α = 0.79).

##### Sexual IPV

Respondents were asked how often their previous partner had used sexual violence across four items (forced them to have sexual intercourse by holding them down or hurting them, attempted to force intercourse, made them take part in any kind of unwanted sexual activity or being unable to refuse, consented sexual activity because they were afraid of what the partner might do if they refused). Category responses ranged from 1 (*never*) to 4 (*6 or more times*). Average sexual violence from previous partner was *M* = 1.13 (SD = 0.49) (Cronbach’s α = 0.89).

##### Psychological difficulties

Foa et al. (2000) operationalized this construct using information about posttraumatic stress disorder (PTSD), depression, anxiety, and substance abuse. Only information about depression and anxiety was available in the FRA survey. Respondents were asked whether they have suffered depression and/or anxiety as a result of the more serious IPV incidents with a previous partner. Category responses were 0 = No, and 1 = Yes. Psychological difficulties scores were calculated summing all the Yes responses (*M* = 0.42, SD = 0.65).

##### Children witnessing IPV

Respondents were asked whether have any children who have been living with her ever been aware of any violent incidents by the previous partner. Category responses were: 1, Yes, 2, No, and 3, No children living with me at the time of the incidents. Original responses were coded as 1, Yes (1836, 77.3%) and 0, No or no children living with her (540, 22.7%).

##### Ending the relationship because of IPV

Participants were asked whether they ended the relationship because of violence: Did you end your relationship with any of your previous partners because of violence? Category responses were: (1) Yes, the main reason (55.0%, 962); (2) Yes, but it was not the main reason (20.4%, 356); and (3) No (24.6%, 430). We coded this variable to have the value of 1 for Yes, the main reason, and the value of 0 for all the remaining responses.

#### Current Relationships

##### IPV with the current partner

Respondents informed to the interviewer whether they had experienced psychological, physical, or sexual violence with the current partner (different from the actual partner).

##### Physical IPV

Respondents were asked how often their current partner had used physical violence toward them across six items (pushed them, slapped them, thrown hard objects at them, grabbed or pulled their hair, beat them with a fist or a hard object, or kicked them). Category responses ranged from 1 (*never*) to 4 (*all the time*). Average physical violence from current partner was *M* = 1.06 (SD = 0.27).

##### Psychological IPV

Respondents were asked how often their current partner had used psychological violence across four items (belittled or humiliated them in front of other people or in public, scared or intimidated them on purpose, made them watch pornographic material against their wishes, or threatened to hurt them physically). Category responses ranged from 1 (*never*) to 4 (*6 or more times*). Average psychological violence from current partner was *M* = 1.09 (SD = 0.27).

##### Sexual IPV

Respondents were asked how often their current partner had used sexual violence across four items (forced them to have sexual intercourse by holding them down or hurting them, attempted to force intercourse, made them take part in any kind of unwanted sexual activity or being unable to refuse, consented sexual activity because they were afraid of what the partner might do if they refused). Average sexual violence from current partner was *M* = 1.02 (SD = 0.15). A single measure of IPV with the current partner was calculated averaging the scores of psychological, physical, and sexual IPV with the current partner (Cronbach’s α = 0.81; *M* = 1.08; SD = 0.21). Those respondents scoring 1 (never) were classified as resilient women while those women scoring higher than 1 were classified as non-resilient women.

##### Length of the current relationship

Respondents were asked: How long have you been together in total since you started dating? Category responses ranged from 1, under a year to 7, more than 50 years. Averaged length was close to 11–20 years (*M* = 2.96, SD = 1.076).

#### Non-partner Victimization

##### Victimization by non-partners

Respondents were asked about having experienced physical and sexual violence with other adults than partners or ex-partners. The same scales of physical (*M* = 1.27, SD = 0.52) and sexual (*M* = 1.08, SD = 0.29) IPV were used referring to people other than partner or ex-partners (Cronbach’s α = 0.79).

#### Child Abuse

##### Child abuse (CA)

Respondents were asked how often they had experienced different types of physical, emotional or sexual acts from somebody older than 18 years when they were under 15 years of age.

##### Physical CA

The following five items were used to measure physical CA: (1) slap you or pull your hair so that it hurt you, (2) hit you very bad so that it hurt you, (3) kick you very bad so that it hurt you, and (4) beat you very bad with an object like a stick, cane or belt so that it hurt you, and (5) stub or cut you with something so that it hurt you. Category responses ranged from 1 (never) to 3 (more than once).

##### Emotional CA

The interview used the following four items to measure emotional CA: (1) say you that you were not loved, (2) say you that they wished you had never been born, (3) threaten to abandon you or throw you out of the family home, and (4) threaten to hurt you badly or kill you. Category responses ranged from 1 (never) to 3 (more than once).

##### Sexual CA

The interview used the following five items to measure sexual CA: (1) expose their genitals to you when you did not want them to, (2) make you pose naked in front of any person or in photographs, video or an internet webcam; (3) Touch your private parts – genitals or breasts – when you did not want them to, (4) make you touch their private parts – genitals or breasts – when you did not want to, and (5) Make you have sexual intercourse with them when you did not want to. Category responses ranged from 1(never) to 3 (more than once). A single measure of CA was calculated averaging the scores of physical, emotional, and sexual CA (Cronbach’s α = 0.73; *M* = 1.15; SD = 0.25).

#### Country-Level Variable

##### Human development index (HDI)

The HDI measures country development by combining health, education, and wealth. A higher value indicates a higher level of human development (*M* = 871.54, SD = 35.40). The HDI information for each country was retrieved from international databases (United Nations Development Programme, 2013). Other research has also incorporated HDI as a variable of the country-level and has found that this index outperforms other indexes such as the Gender Inequality Index in its relationship with both IPV and acceptability of IPV (Herrero et al., 2017a).

#### Sociodemographic Characteristics

The survey provided data about respondents age, satisfaction with household income, size of locality, and educational background.

##### Age

The ages of respondents were originally coded into seven age groups: 18–24, 25–29, 30–34, 35–39, 40–49, 50–59, and 60+ (*M* = 5.06, SD = 1.503). The average respondent was in the 40–49 age group.

##### Dissatisfaction with household income

The respondents’ satisfaction with household income was measured with the following question: “Which one of the descriptions on this card comes closest to how you feel about your household income nowadays?” Responses were coded from 1 (living comfortably on present income) to 4 (finding it very difficult on present income; *M* = 2.16, SD = 0.95).

##### Size of locality

Respondents were asked to describe the type of locality in which they lived: “Which option on this card best describes the area where you live in?” The responses were coded from 1 (a big city or outskirts of a big city) to 4 (a farm or home in the countryside; *M* = 2.69, SD = 1.21).

##### Educational background

Respondents’ educational background was coded using a three-category response scale in the original dataset to make the results comparable across different national educational systems from 1 (primary) to 3 (tertiary; *M* = 1.93, SD = 0.70).

#### Interviewer Variables

##### Insincere responses

Interviewers were asked to assess whether each respondent’s responses were insincere overall: “Do you think the respondent was telling the truth in the interview?” Responses ranged from 1 (*yes, all the time*) to 4 (*not at all*; *M* = 1.19, SD = 0.46). Although other research with this same dataset has also controlled for privacy and safety during the interview (Herrero et al., 2017b), we did not include these variables in the analyses because of zero variance in some countries (Germany, Lithuania, Netherlands, Portugal, Sweden, and United Kingdom).

### Statistical Procedures

We used multilevel regression modeling to take into account the hierarchical structure of the data – individuals (level 1) nested within countries (level 2). All predictors were centered around the grand mean to ease interpretation of results. Multiple imputations of missing values were performed (Rubin, 1996).

## Results

**Table 1** shows the results of the multilevel regression model. At the individual level, age, satisfaction with household income, length of the current relationship, psychological difficulties, physical IPV with previous partner, child abuse, victimization with other people than the partner or ex-partner, and having ended a previous relationship because of IPV showed a significant relationship with the groups of non-resilient and resilient women. Age was positively related to being in the group of resilient women: a one-unit increase in age increased 1.11 times the odds of being in the group of resilient women. Dissatisfaction with household income (*b* = -0.213, *p* < 0.01) was negatively related to being in the group of resilient women. Given that this variable takes the value 0 for non-resilient women and the value 1 for resilient women, dissatisfaction with household income is predictive of being in the group of non-resilient women. When the OR is lower than 1 and significant, as with dissatisfaction with household income (OR = 0.808), the inverse (1/OR = 1/0.808 = 1.24) measures the association between the predictor and lower values on the grouping variable (being in the non-resilient group of women). Thus, a one-unit increase in dissatisfaction with income increased 1.24 times the odds of being in the group of non-resilient women. Length of the current relationship (OR = 0.759, inverse = 1.32) was also significantly associated with the outcome variable. A one-unit increase in the length of the current relationship increased 1.32 times the odds of being in the group of non-resilient women.

**Table 1:** Non-resilient vs. resilient women to intimate partner violence (IPV) in the European Union (*N* = 2376): Unstandardized multilevel regression estimates, robust standard errors, and 95% confidence intervals (C.I.).

**Parameter**		**Estimate**	**95% C.I.**	**Odds ratios [95% C.I.]**
		
Threshold		–1.087 (0.073)^∗∗∗^	[–1.207, –0.967]	
Individual-level				
	Insincerity	–0.363 (0.131)**	[–0.579, –0.148]	0.695 [0.560, 0.862]
	Age	0.107 (0.048)*	[0.028, 0.186]	1.113 [1.029, 1.205]
	Dissatisfied with income	–0.213 (0.067)**	[–0.323, –0.103]	0.808 [0.724, 0.902]
	Size of locality	0.007 (0.063)	[–0.097, 0.111]	1.007 [0.908, 1.118]
	Educational background	0.094 (0.089)	[–0.053, 0.241]	1.098 [0.948, 1.273]
	Length of relationship	–0.275 (0.076)***	[–0.401,– 0.150]	0.759 [0.670, 0.861]
	Previous psychological IPV	0.082 (0.177)	[–0.208, 0.373]	1.086 [0.812, 1.452]
	Previous physical IPV	0.393 (0.102)***	[0.226, 0.561]	1.482 [1.254, 1.752]
	Previous sexual IPV	0.031 (0.081)	[–0.102, 0.165]	1.032 [0.903, 1.179]
	Psychological difficulties	–0.182 (0.086)*	[–0.324, –0.041]	0.833 [0.724, 0.960]
	Children witnessing IPV	–0.139 (0.126)	[–0.346, 0.067]	0.870 [0.708, 1.070]
	Child Abuse	–1.215(0.245)***	[–1.618, –0.812]	0.297 [0.198, 0.444]
	Adult victimization with non-partners	–0.628 (0.179)***	[–0.922, –0.334]	0.534 [0.398, 0.716]
	Ended relationship because of violence	0.355 (0.135)**	[0.133, 0.577]	1.426 [1.142, 1.781]
Country-level				
	Human development index (HDI)	0.005 (0.002)**	[0.002, 0.009]	
Residual variance		0.061 (0.030)*	[0.011, 0.111]	

*^∗^p < 0.05, ^∗∗^p < 0.01, ^∗∗∗^p < 0.001.*

Physical IPV with the previous partner was positively associated with the outcome variable (OR = 1.48): a one-unit increase in physical IPV from the previous partner increased 1.48 times the odds of being in the resilient group of women. Psychological difficulties stemming from IPV was associated with the outcome variable (OR = 0.833, inverse = 1.20): a one-unit increase in psychological difficulties increased 1.20 the odds of being in the non-resilient group of women. A larger statistical relationship was found for child abuse (*b* = -1.215, *p* < 0.001) and victimization by non-partners (*b* = -0.628, *p* < 0.001). Looking at the inverse of their odds ratios, a one-unit increase in child abuse (OR = 0.297, inverse = 3.37) increased 3.37 times the odds of being in the group of non-resilient women. Transforming the odds ratio (0.297) to probabilities (odds/odds+1 = 0.23) gives an intuitive illustration of the effect of child abuse on IPV victimization from multiple partners. Although by chance women of the sample would have a probability of 50% to be in the resilient group, a one-unit increase in child abuse reduces this probability in 27% (0.50–0.23 = 0.27). A one-unit increase in victimization by non-partners (OR = 0.534, inverse = 1.87) increased 1.87 times the odds of being in the group of non-resilient women. Having ended a previous relationship because of IPV significantly increased the odds of being in the resilient group of women (OR = 1.426).

As for the country-level variable of the study, the unstandardized coefficient expresses the linear relationship between HDI and the ratio resilient/non-resilient women in each country. Note at this point that, while the dependent variable is dichotomous at the individual level (non-resilient vs. resilient), it is no longer dichotomous at the country level. This is so because for each country the ratio of resilient/non-resilient women is estimated and this ratio is no longer dichotomous, but continuous. Higher values in country HDI were statistically related (*b* = 0.005, *p* < 0.01) to a higher proportion of resilient women in that country (higher values in the outcome variable). In other words, countries higher on HDI show a tendency to have a greater proportion of resilient women than the average country in terms of HDI.

Overall, when compared to non-resilient women, resilient women were more satisfied with household income, had shorter current relationships, informed to the interviewer to have experienced higher levels of previous physical IPV, as well as fewer child abuse and fewer victimization by non-partners or ex-partners, and experienced less psychological difficulties as a result of previous IPV. They ended the relationship because of the violence to a greater extent and lived in countries ranked higher in HDI. These results take into account the hierarchical structure of the data and are adjusted by the insincerity of respondents as appraised by the interviewer. In fact, this variable turned out to be statistically significant (*b* = -0.363, *p* < 0.10, OR = 0.695). If we consider the inverse of the OR (= 1/0.695 = 1.43) we see that a one-unit increase in insincerity increased 1.43 times the odds of being in the group of non-resilient women. In other words, the women who reported IPV for multiple partners were evaluated by the interviewers as less sincere.

## Discussion

The study of the multiple IPV victimization by different partners is a relatively neglected area of study. The absence of a greater research effort in this area is surprising considering the frequency with which some women are victimized by different partners (Krause et al., 2006). In the present study, we aimed to analyze the differences between non-resilient (multiple IPV victimization) and resilient (non-multiple IPV victimization) women from the 28 member countries of the European Union (*N* = 2376). Based on the available literature and empirical evidence we included the study of a number of variables about women past victimization by both partners and non-partners, child abuse, psychological consequences of the abuse, and socio-demographic characteristics. Additionally, we also included variables that have not been traditionally analyzed in this area. Specifically, we also studied the influence of the victim’s ability to end the abusive relationship -at the individual level- and the human development of the country in which the women lives -at the country level.

Some of the results obtained in other studies were replicated in our study. With regard to sociodemographic variables, we found, as well as other studies, that victimization by different partners is more frequent among younger women (Testa et al., 2003; Alexander, 2009; Tsirigotis and Łuczak, 2018). We also found that non-resilient women had lower satisfaction with income than resilient women. The evidence available at this point in the literature is mixed. In the review of studies by Ørke et al. (2018), only one study reported a positive relationship between low income and victimization by multiple partners (Vatnar and Bjørkly, 2008) while other research on IPV has informed of a negative relationship between satisfaction with income and both physical and psychological IPV (Herrero et al., 2017b).

Length of the relationship was positively related to IPV victimization by multiple partners. As Logan et al. (2006) suggested, during the first stages of a new relationship it might be not only that IPV is absent but also that IPV cues are misinterpreted (e.g., controlling, monitoring, and stalking behaviors). If this is the case, as the relationship progresses with time, women may be able to correctly identify episodes of IPV with their partner.

An important aspect that the present investigation has not been able to confirm is the relationship between previous and actual IPV. There are some studies that have found that higher levels of previous IPV were predictive of higher current IPV (Testa et al., 2003; Cole et al., 2008) while others have not found a significant association (Stein et al., 2016). Our results are partially in the same line as those found by Stein et al. (2016) since both psychological and sexual IPV with the previous partner was unrelated to current IPV. Our study found a negative relationship between previous physical IPV and current IPV, however, those women who had suffered more physical IPV with previous partners were more resilient to future IPV, as they more frequently informed to the interviewer of a free-IPV relationship with their current partner. According to Foa et al’s. (2000) model, women who experience more severe IPV, as well as higher levels of psychological difficulties, become more vulnerable, thus reducing their resiliency to future IPV. What we found in our study is that resilient women suffered less psychological difficulties as a consequence of the abuse, as predicted by Foa et al. (2000), but they also presented higher levels of previous physical IPV. In this same line is the work of Cobb et al. (2006) who found greater post-traumatic growth in women with higher rates of previous physical IPV, which suggests that these women with higher rates of previous physical IPV had managed to overcome the violence. It seems, therefore, that some women with higher levels of previous physical IPV reacted to the abuse in a way that reduced their psychological vulnerability, which in turn translated into greater resilience.

One of the possible victim’s responses with a detrimental effect on future IPV could be ending the relationship due to the violence suffered (Cobb et al., 2006). The resilient women in our study reported having ended the relationship due to episodes of violence more frequently than non-resilient women. The way these women cope with the abuse seems, therefore, more relevant to their personal adjustment than the levels of IPV suffered (Senter and Caldwell, 2002). This would be especially relevant in the case of previous physical IPV, as our data suggest. These results complement those obtained in the study of repeated violence with the same partner. According to Dichter and Gelles (2012), the evidence on the effect of breaking the abusive relationship in the subsequent rates of IPV could be explained in terms of the reasons underlying that violence, differentiating what is battering from what it is not. When the violence exerted is motivated to gain coercive control over the victim (battering), victim’s decisions about ending the relationship could exacerbate these violent episodes in the aggressor. Thus, leaving would threaten the aggressor’s dominance, which may use violence in an attempt to regain power. Alternatively, when the violence exercised is mainly motivated by anger, frustration, retaliation, or self-defense, the end of the relationship is likely to lead to the cessation of violence. As pointed out by Dichter and Gelles (2012), the measurement of IPV from violent incidents, as in the present study, does not allow to differentiate battering from what is not, since the former must also include an evaluation of the dynamic of the violence in the relationship. This should include aspects such as the perpetrator’s motive to control the victim or the victim’s experience of being dominated and controlled by the violence. Future research on the victimization of IPV by multiple partners should, therefore, include measures on the reasons for violence to identify the existence of battering to further verify this assumption. This might shed light on to what extent the rupture of the violent relationship may or may not remain a potential risk situation for the victim.

The strongest predictor of IPV victimization by multiple partners found in our study was the level of child abuse suffered. This may be because victims of child abuse tend to associate with potentially abusive partners in adulthood. This process, which Herrero et al. (2018) have called conditional partner selection, suggests that victims of abuse in childhood develop a series of psychological deficits that increase the likelihood of ending with potentially abusive partners (see Torres et al., 2013 for analysis of the characteristics of potentially abusive partners), who also find some of these deficits as something attractive in their partners (i.e., anxiety attachment). The empirical link between child abuse and IPV has been consistently found in a number of studies (see an analysis in Herrero et al., 2018). Likewise, in their review of studies on victimization by multiple partners, Ørke et al. (2018) only found one study that did not find any positive relationship between child abuse and victimization by multiple partners (Coolidge and Anderson, 2002) and one study (Alexander, 2009) that only found this link for child sexual victimization but not for abuse and neglect. Our results add to this empirical evidence and help to situate adverse experiences in childhood as one of the main predictors of IPV victimization by different partners in adulthood. This evidence also adds to the empirical evidence already found between child abuse and adulthood victimization.

Adult victimization by adults other than partner and ex-partner was also found to be an important predictor of IPV victimization by different partners. Although its effect is less than that found for child abuse, its influence is relevant when explaining the IPV victimization by different partners. The literature on the effects of lifetime victimization on partner abuse has found mixed results depending on the type of adult victimization measured (Cole et al., 2008; Stein et al., 2016). For instance, Stein et al. (2016) did not find a statistical relationship between having been a victim of both sexual and non-sexual assault and multiple victimizations by different partners in 164 women victims of IPV (Stein et al., 2016). The relationship between non-IPV and multiple IPV victimization by different partners in adulthood has been traditionally explained in terms of the increase in the vulnerability of women victims of victimization and their problems with interpersonal relationships (Nishith et al., 2000; Classen et al., 2005). From this point of view, victimization in adulthood would operate in a similar way to how childhood victimization operates: decreasing the psychological resources of the victim and negatively conditioning their social development (Herrero et al., 2018). Because victimization in childhood has a greater effect on the victimization of IPV by multiple partners than non-IPV adult victimization, according to our findings the sooner victimization occurs, the greater will be its effect on the psychological and social development of the victim.

At the country level, our results suggest that the greater the human development of a country, the higher the proportion of women resilient to IPV in this country. This result points to the importance of the structural factors to understand both the IPV and the IPV victimization by different partners. While the effect of structural factors on IPV is being studied in recent years, this research effort has not seemed to be transferred to the study of IPV victimization by different partners. Herrero et al. (2018) found among more than 20,000 women of 28 countries of the European Union that the country’s human development not only negatively influenced the country’s IPV rates but it also affected the way in which other predictors of the IPV operated. Other research had already shown a few years earlier that the structural conditions of societies could affect their gender value systems. Thus, citizens from countries with more egalitarian structural conditions (measured through indexes such as the Gender Empowerment Index or the HDI) tend to show more egalitarian gender attitudes and lower IPV acceptability (Gracia and Herrero, 2006; Brandt, 2011). Our results allow extending the influence of the structural conditions not only to women’s IPV victimization but to the victimization by different partners. Citizens of countries higher on human development are not only more protected against potential IPV victimization but also tend to be more resilient to this IPV. This circumstance, undoubtedly, suggests extending preventive efforts to levels other than those of the individual. Future research should also incorporate the study of contextual factors since they not only influence the rates of IPV but may also influence other risk factors observed in this study. Contextual risk factors associated to child abuse (Gracia and Herrero, 2008) are particularly relevant at this point, since it has shown to be one of the most important predictors of resilience to multiple IPV victimization by different partners in our study.

The fact that the main antecedent of the multiple victimization found in our study – the child abuse suffered – is distal in nature, should make us think about the importance of global preventive policies throughout the life cycle. Adverse experiences in childhood negatively affect the personal and social development of women, and the structural conditions of the society in which they live exert a notable influence as well. These adverse experiences in childhood not only condition violent relationships with specific partners. Rather, they seem to be linked to a trajectory of sustained vulnerability in intimate partner relationships, characterized by repeated victimization with different partners. In addition, the structural conditions, which are beyond the control of the victim of IPV, also have a distal nature. Again at this point, preventive policies and an orientation of the public administrations toward an improvement of the human development level of the countries are needed.

### Strengths and Limitations

The present study presents strengths as well as potential limitations. Among the strengths we highlight, on the one hand, the sample used. Having a large sample of IPV resilient and non-resilient women from a probabilistic sample of women from the 28 countries of the European Union is a strength of the study. The lack of large representative samples has probably been a limitation to the development of research in this area. The present study allows an approximation of the real percentage of IPV resilient women in the European Union, which is estimated at around 73% of the female population that has previously suffered IPV with other partners. According to this, a large percentage of the women who suffered IPV were in some way resilient to IPV and able to avoid new violent relationships. The absence of psychological difficulties and the lower frequency of adverse situations (in childhood or in adulthood) are among the characteristics of this group of resilient women. On the other hand, the multi-country nature of the sample has allowed the analysis of structural influences on resilience, which constitutes an innovation in this field of study. Although it is increasingly common to incorporate the study of the influence of structural factors on IPV, to our knowledge this has not yet been applied to the study of IPV resilience. Structural explanations are important since they allow to effectively contextualize the processes under study. If IPV is conditioned by structural factors of society such as the gender value system or gender-related inequalities in health, education, economy, or politics, it is advisable to study them in investigations that incorporate, as the present one, variables in the individual and country levels.

The present investigation, however, is not free of potential limitations. A first limitation would be the fact that the FRA survey does not include information on the behavior of the woman and therefore does not allow an analysis of the bidirectionality of the violence. An alternative explanation for the lack of resiliency of some women in the study could be their aggressive behavior that might elicit aggressive responses in their partners. It would not be exclusively, therefore, a matter of victimization, but also of perpetration of the IPV. Unfortunately, we do not have this information, so further studies should aim to identify this potential group of more aggressive women so as not to confuse them with non-resilient women. Related to this, the interview was the only measure administered and, although controls were carried out on potential response biases, future research would benefit from the inclusion of additional measures beyond the context of the interview. Of particular importance would be the assessment of the mental health status of the participants to potentially exclude women with mental illness whose responses may be distorted, which would pose a threat to the validity of the study. The controls on the response bias performed in the analyses, however, could have alleviated this potential threat. Another possible limitation may lie in the retrospective nature of this work: the victimization rates in the adulthood could have conditioned the recall of victimization episodes suffered in childhood. Although this is a possibility that is always present in retrospective studies that involve remembering past situations that are sometimes very distant in time, the relationships observed between victimization in childhood and in adulthood in this study are consistent with what has been obtained in other investigations (Herrero et al., 2018). It does not seem therefore that the nature of the study has substantially affected the results of the study.

The present investigation has allowed knowing in greater depth some risk factors of women non- resilient to the IPV. Some aspects related to the resilience and that could also exert a notable influence were not considered, however. For example, the empirical evidence indicates that social support is negatively related both to the repeated violence of IPV and to the victimization of IPV by different partners (Kuijpers et al., 2011; Dichter and Gelles, 2012) and that it can be an important characteristic of resilient women (Dutton and Greene, 2010). Further, as most research on social support and resilience has focused primarily on social support from families and friends (Norris and Stevens, 2007), research in this area would benefit from the inclusion of areas of social support other than family and friends that have been linked to resilience stemming from the community (Norris and Stevens, 2007), such as community integration, and both formal and informal community support (Herrero et al., 2004, 2011; Herrero and Gracia, 2007; Juarros-Basterretxea et al., 2018).

## Ethics Statement

All subjects gave written informed consent in accordance with the Declaration of Helsinki. Ethics approval for the secondary analysis of data was obtained from the Agency for Fundamental Rights of the European Union, who provided a special license for this purpose (Reference No. 96457). Also, the study is compliant with the statements of Principle in Code of Practice for Official Statistics and the specific requirements of the Protocol on Data Access and Confidentiality of the United Kingdom, where the UK Data Archive stores the data.

## Author Contributions

All authors contributed equally to this manuscript. It was based on an original draft of JH, further improved by PV, AT, and FR in terms of conceptual framing, data analysis, and discussion of results.

## Conflict of Interest Statement

The authors declare that the research was conducted in the absence of any commercial or financial relationships that could be construed as a potential conflict of interest.
